# The Complete Chloroplast Genome Sequence of the Medicinal Plant *Salvia miltiorrhiza*

**DOI:** 10.1371/journal.pone.0057607

**Published:** 2013-02-27

**Authors:** Jun Qian, Jingyuan Song, Huanhuan Gao, Yingjie Zhu, Jiang Xu, Xiaohui Pang, Hui Yao, Chao Sun, Xian’en Li, Chuyuan Li, Juyan Liu, Haibin Xu, Shilin Chen

**Affiliations:** 1 The National Engineering Laboratory for Breeding of Endangered Medicinal Materials, Institute of Medicinal Plant Development, Chinese Academy of Medical Sciences and Peking Union Medical College, Beijing, China; 2 Guangzhou Pharmaceutical Holding Limited, Guangzhou, China; 3 Institute of Chinese Materia Medica, China Academy of Chinese Medicinal Sciences, Beijing, China; National Taiwan University, Taiwan

## Abstract

*Salvia miltiorrhiza* is an important medicinal plant with great economic and medicinal value. The complete chloroplast (cp) genome sequence of *Salvia miltiorrhiza*, the first sequenced member of the Lamiaceae family, is reported here. The genome is 151,328 bp in length and exhibits a typical quadripartite structure of the large (LSC, 82,695 bp) and small (SSC, 17,555 bp) single-copy regions, separated by a pair of inverted repeats (IRs, 25,539 bp). It contains 114 unique genes, including 80 protein-coding genes, 30 tRNAs and four rRNAs. The genome structure, gene order, GC content and codon usage are similar to the typical angiosperm cp genomes. Four forward, three inverted and seven tandem repeats were detected in the *Salvia miltiorrhiza* cp genome. Simple sequence repeat (SSR) analysis among the 30 asterid cp genomes revealed that most SSRs are AT-rich, which contribute to the overall AT richness of these cp genomes. Additionally, fewer SSRs are distributed in the protein-coding sequences compared to the non-coding regions, indicating an uneven distribution of SSRs within the cp genomes. Entire cp genome comparison of *Salvia miltiorrhiza* and three other Lamiales cp genomes showed a high degree of sequence similarity and a relatively high divergence of intergenic spacers. Sequence divergence analysis discovered the ten most divergent and ten most conserved genes as well as their length variation, which will be helpful for phylogenetic studies in asterids. Our analysis also supports that both regional and functional constraints affect gene sequence evolution. Further, phylogenetic analysis demonstrated a sister relationship between *Salvia miltiorrhiza* and *Sesamum indicum*. The complete cp genome sequence of *Salvia miltiorrhiza* reported in this paper will facilitate population, phylogenetic and cp genetic engineering studies of this medicinal plant.

## Introduction

Chloroplasts, one of the main distinguishing characteristics of plant cells, are now generally accepted to have originated from cyanobacteria through endosymbiosis [1], [2]. In addition to their central function of photosynthesis, chloroplasts also participate in the biosynthesis of starch, fatty acids, pigments and amino acids [3]. Since the first cp genome sequence of *Marchantia polymorpha* [4] was reported in 1986, over 285 complete cp genome sequences have been deposited in the NCBI Organelle Genome Resources (www.ncbi.nlm.nih.gov/genomes/ORGANELLES/organelles.html). A typical circular cp genome has a conserved quadripartite structure, including a pair of inverted repeats (IRs), separated by a large single-copy region (LSC) and a small single-copy region (SSC). In angiosperms, the majority of the cp genomes range from 120 to 160 kb in length [5] and exhibit highly conserved gene order and contents [2], [6]. However, large-scale genome rearrangement and gene loss have been identified in several angiosperm lineages [7], [8]. Cp genome sequences are useful for phylogenetic [9], DNA barcoding [10], population [11] and transplastomic [12] studies.

*Salvia miltiorrhiza* Bunge (Danshen in Chinese) is a deciduous perennial flowering plant in the family Lamiaceae and the order Lamiales. It is a significant traditional Chinese medicinal herb widely cultivated in China with great economic and medicinal value [13]. The dried roots of *Salvia miltiorrhiza*, commonly known as ‘Chinese sage’ or ‘red sage’ in western countries, are widely used in the treatment of several diseases, including but not limited to cardiovascular, cerebrovascular and hyperlipidemia diseases [14]–[17]. More than 70 compounds have been isolated and structurally identified from the root of *Salvia miltiorrhiza* to date [18], [19]. These compounds can be divided into two major groups: the hydrophilic phenolic acids, including rosmarinic, lithospermic and salvianolic acids; and the lipophilic components, including diterpenoids and tanshinones [14], [19]. Modern pharmacological research has demonstrated that compounds in both categories have multiple important and desirable therapeutic actions, including antitumor, anti-inflammatory, antimicrobial, antivirus, anti-atherosclerotic and antioxidant activities [14], [15], [20]. In addition to the significant medicinal value described above, *Salvia miltiorrhiza* is exemplary for its relatively small genome size (∼600 Mb), short life cycle and genetic transformability [21]–[24]. These characteristics make *Salvia miltiorrhiza* an exemplary starting point to investigate the mechanism of medicinal plant secondary metabolism.

To date, few data are available regarding the *Salvia miltiorrhiza* cp genome. Here, as a part of the genome sequencing project of *Salvia miltiorrhiza*, we report its complete cp genome sequence, determined using both pyrosequencing and SOLiD technologies. To the best of our knowledge, this is the first complete cp genome sequence in Lamiaceae, the sixth-largest family of angiosperms [25]. Comparative sequence analysis was conducted among published asterid cp genomes. These data may contribute to a better understanding of evolution within the asterid clade.

## Materials and Methods

### DNA Sequencing, Genome Assembly and Validation

Fresh leaves were collected from the *Salvia miltiorrhiza* Bunge (line 993) grown in a field nursery at the medicinal plant garden of the Institute of Medicinal Plant Development. Total DNA was extracted using the DNeasy Plant Mini Kit (Qiagen, CA, USA) and used for constructing shotgun libraries according to the manufacturer’s manual for the 454 GS FLX Titanium [26]. A total of 20 GS FLX runs were carried out for the project. In addition, three 2×50 mate-paired libraries with insert sizes of 1, 3 and 5 kb were constructed following the SOLiD Library Preparation Guide and sequenced on a SOLiD 3 plus platform for 1/2, 3/4 and 1/2 runs, respectively.

After quality control, the trimmed and cleaned reads were used to assemble the cp genome. First, the 454 reads were used to generate a raw cp genome assembly. Then, the SOLiD mate-paired reads were mapped to the raw assembly using BioScope (version 1.3, see BioScope Software for Scientists Guide) to correct the erroneous homopolymers. We thus acquired a high quality complete cp genome. To verify the assembly, four junction regions between IRs and LSC/SSC were confirmed by PCR amplifications and Sanger sequencing using the primers listed in Table S1.

### Genome Annotation, Codon Usage and Intra-specific SNPs

The cp genome was annotated using the program DOGMA [27] coupled with manual corrections for start and stop codons. The tRNA genes were identified using DOGMA and tRNAscan-SE [28]. The nomenclature of cp genes was referred to the ChloroplastDB [29]. The circular cp genome map was drawn using the OGDRAW program [30]. Codon usage and GC content were analyzed using MEGA5 [31]. Intra-specific SNPs were called by mapping the SOLiD mate-paired reads to the cp genome assembly using BioScope.

### Genome Comparison and Repeat Content

MUMmer [32] was used to perform pairwise cp genomic alignment. mVISTA [33] was used to compare the cp genome of *Salvia miltiorrhiza* with three other cp genomes using the annotation of *Salvia miltiorrhiza* as reference. REPuter [34] was used to visualize both forward and inverted repeats. The minimal repeat size was set to 30 bp and the identity of repeats was no less than 90% (hamming distance equal to 3). Tandem repeats were analyzed using Tandem Repeats Finder (TRF) v4.04 [35] with parameter settings as described by Nie et al [36]. Simple sequence repeats (SSRs) were detected using MISA (http://pgrc.ipk-gatersleben.de/misa/), with thresholds of eight repeat units for mononucleotide SSRs, four repeat units for di- and trinucleotide SSRs and three repeat units for tetra-, penta- and hexanucleotide SSRs. All of the repeats found were manually verified, and the redundant results were removed.

### Sequence Divergence and Phylogenetic Analysis

The 29 complete cp sequences representing the asterid lineage of angiosperms were downloaded from NCBI Organelle Genome Resources database (Table S2). The 80 protein-coding gene sequences were aligned using the Clustal algorithm [37]. Pairwise sequence divergences were calculated using Kimura’s two-parameter (K2P) model [38].

For the phylogenetic analysis, a set of 71 protein-coding genes commonly present in the 30 analyzed genomes was used. Maximum parsimony (MP) analysis was performed with PAUP*4.0b10 [39] using heuristic search, random addition with 1,000 replicates and tree bisection-reconnection (TBR) branch swapping with the Multrees option in effect. Bootstrap analysis was performed with 1,000 replicates with TBR branch swapping. Maximum likelihood (ML) analysis was also performed using PAUP with the GTR+I+G nucleotide substitution model. This adopted best-fit model was determined by Modeltest 3.7 [40]. *Spinacia oleracea* and *Arabidopsis thaliana* were set as outgroups.

## Results

### Genome Assembly and Validation

The annotated cp genome sequence of *Arabidopsis thaliana* was taken from TAIR (http://www.arabidopsis.org/). The *Arabidopsis* genes encoding *psbA* and *ndhI* were located in the LSC and SSC regions of the cp genome, respectively. Homologs of these two genes were identified in the *Salvia miltiorrhiza* cp genome by searching 454 reads using the BLASTn algorithm [41]. Both genes then served as seed sequences for *Salvia miltiorrhiza* cp genome assembly.

The draft sequence of the *Salvia miltiorrhiza* cp genome was constructed by extending the two seed sequences on both the 5′ and 3′ ends in a step-by-step manner until they overlapped at both the IRa and IRb regions. Detailed procedures for each extension step are described as follows. All of the 454 reads showing homology to the seed sequence were identified in a similarity search, using the BLASTn algorithm with a threshold of ≥95% homology. Of these reads, the one with best alignment to the 5′ or 3′ end of the seed sequence was selected and used to extend the seed sequence.

Cp genome reads were screened out by mapping all 454 reads to the draft cp genome sequence, using the BLASTn algorithm with a threshold of ≥95% homology. A total of 1,767,159 reads (7.4% of total reads) were obtained, with an average length of 384 bp, thus yielding 4,492× coverage of the cp genome. The consensus sequence for a specific position was generated by assembling reads mapped to the position using CAP3 [42] and was then used to construct the complete sequence of the *Salvia miltiorrhiza* cp genome.

Erroneous homopolymers, which are intrinsic to pyrosequencing [43], were manually corrected by mapping all SOLiD reads to the cp genome assembly using BioScope. To validate the assembly, four junctions between IRs and LSC/SSC were confirmed by PCR amplifications and Sanger sequencing. We compared the Sanger results with the assembled genome, and no mismatch or indel was observed, which demonstrated the accuracy of our assembly. The final cp genome of *Salvia miltiorrhiza* was then submitted to GenBank (accession number: JX312195).

### Genome Features

**Figure 1:**
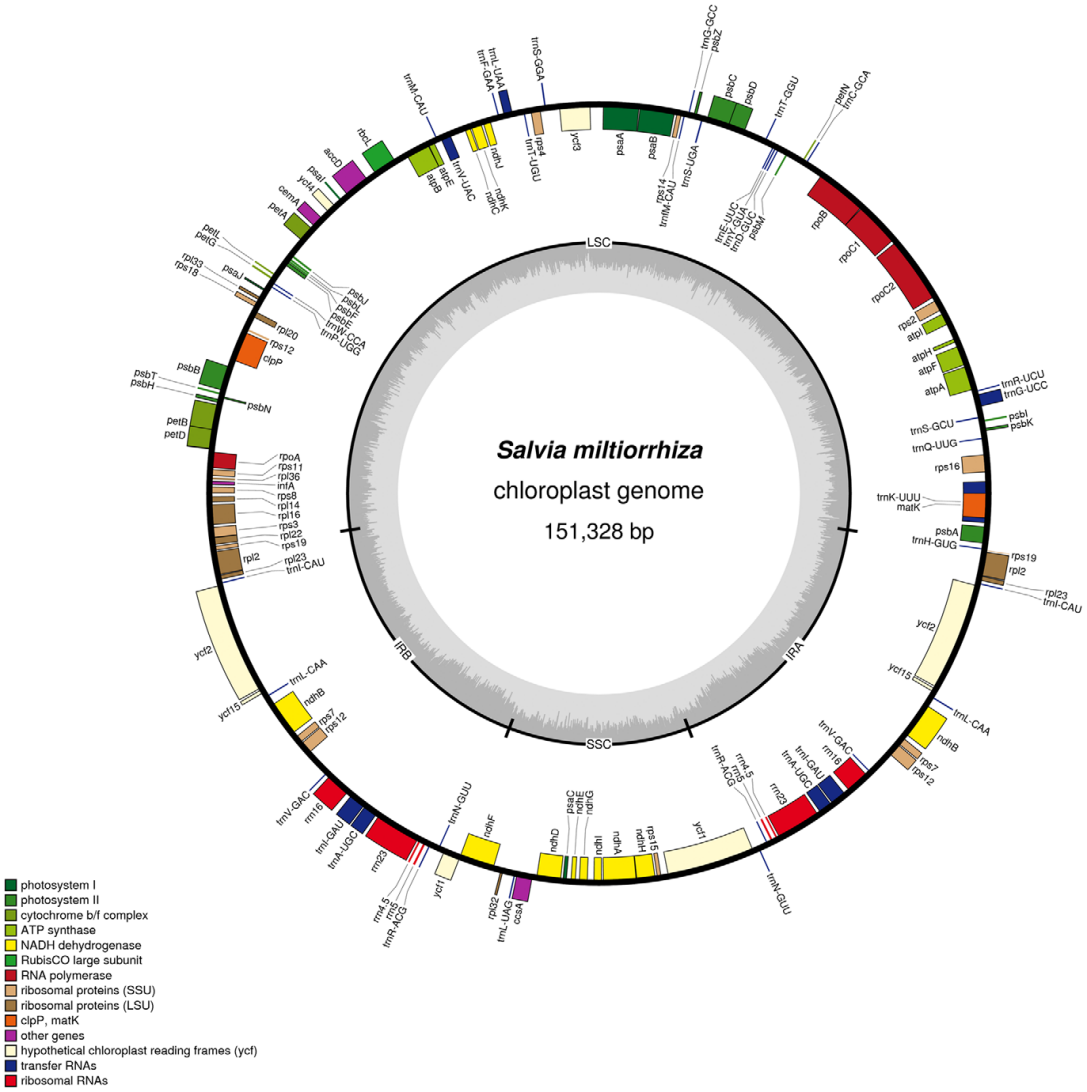
Gene map of the *Salvia miltiorrhiza* chloroplast genome. Genes drawn inside the circle are transcribed clockwise, and those outside are counterclockwise. Genes belonging to different functional groups are color-coded. The darker gray in the inner circle corresponds to GC content, while the lighter gray corresponds to AT content.

The complete cp genome of *Salvia miltiorrhiza* is 151,328 bp in length, which is in range with those from other angiosperms [5], and exhibits a typical quadripartite structure, consisting of a pair of IRs (25,539 bp) separated by the LSC (82,695 bp) and SSC (17,555 bp) regions (Table 1, Figure 1). The overall GC content of the *Salvia miltiorrhiza* cp genome is 38.0%, which is similar to the other reported asterid cp genomes [44]–[48]. The GC content of the IR regions (43.1%) is higher than that of the LSC and SSC regions (36.2% and 32.0%, respectively). The high GC content of the IR regions is caused by the high GC content of the four ribosomal RNA (rRNA) genes (55.2%) present in this region.

**Table 1:** Base composition in the *Salvia miltiorrhiza* chloroplast genome.

		T(U) (%)	C (%)	A (%)	G (%)	Length (bp)
LSC		32.6	18.5	31.2	17.7	82,695
SSC		33.8	16.7	34.2	15.3	17,555
IRa		28.5	22.4	28.4	20.7	25,539
IRb		28.4	20.7	28.5	22.4	25,539
Total		31.3	19.3	30.6	18.7	151,328
CDS		31.4	17.8	30.5	20.3	79,080
	1st position	23.7	19.0	30.5	26.8	26,360
	2nd position	32.6	20.4	29.2	17.8	26,360
	3rd position	37.8	14.0	31.8	16.3	26,360

CDS: protein-coding regions.

The *Salvia miltiorrhiza* cp genome encodes 131 predicted functional genes, of which 114 are unique, including 80 protein-coding genes, 30 transfer RNA (tRNA) genes and four rRNA genes (Figure 1, Table S3). Six protein-coding, seven tRNA and all four rRNA genes are duplicated in the IR regions. The LSC region contains 61 protein-coding and 22 tRNA genes, whereas the SSC region contains 12 protein-coding and one tRNA genes. Similar to the *Nicotiana tabacum* [48] and *Panax ginseng* [47] cp genomes, the *Salvia miltiorrhiza* cp genome has 18 intron-containing genes, 15 (nine protein-coding and six tRNA genes) of which contain one intron and three (*clpP*, *rps12* and *ycf3*) of which contain two introns (Table 2). The *rps12* gene is a trans-spliced gene with the 5′ end located in the LSC region and the duplicated 3′ end in the IR regions. The *trnK-UUU* has the largest intron (2,522 bp) containing the *matK* gene.

**Table 2:** The genes with introns in the *Salvia miltiorrhiza* chloroplast genome and the length of the exons and introns.

Gene	Location	Exon I (bp)	Intron I (bp)	Exon II (bp)	Intron II (bp)	Exon III (bp)
*atpF*	LSC	144	699	411		
*clpP*	LSC	71	692	292	628	228
*ndhA*	SSC	553	985	539		
*ndhB*	IR	777	675	756		
*petB*	LSC	6	702	642		
*petD*	LSC	8	720	475		
*rpl16*	LSC	9	873	399		
*rpl2*	IR	391	658	434		
*rpoC1*	LSC	456	759	1620		
rps12*	LSC	114	-	232	526	26
*rps16*	LSC	42	874	195		
*trnA-UGC*	IR	38	795	35		
*trnG-UCC*	LSC	23	682	48		
*trnI-GAU*	IR	37	940	35		
*trnK-UUU*	LSC	37	2522	35		
*trnL-UAA*	LSC	37	453	50		
*trnV-UAC*	LSC	36	576	37		
*ycf3*	LSC	129	696	228	726	153

The *rps12* is a trans-spliced gene with the 5′ end located in the LSC region and the duplicated 3′ end in the IR regions.

52.3%, 1.8% and 6.0% of the genome sequence encode proteins, tRNAs and rRNAs, respectively. The remaining regions are non-coding sequences, including introns, intergenic spacers and pseudogenes. The 30 unique tRNA genes include all of the 20 amino acids required for protein biosynthesis. Moreover, the 86 protein-coding genes comprise 79,080 bp coding for 26,360 codons. Based on the sequences of protein-coding genes and tRNA genes, the frequency of codon usage was deduced for the *Salvia miltiorrhiza* cp genome and summarized in Table 3. Among these codons, 2,806 (10.6%) encode leucine, and 292 (1.1%) encode cysteine, which are the most and least prevalent amino acids, respectively. Within protein-coding regions (CDS), the percentage of AT content for the first, second and third codon positions are 54.2%, 61.8% and 69.6%, respectively (Table 1). The bias towards a higher AT representation at the third codon position was also observed in other land plant cp genomes [5], [36], [44], [49], [50].

**Table 3:** The codon–anticodon recognition pattern and codon usage for the *Salvia miltiorrhiza* chloroplast genome.

Amino acid	Codon	No.	RSCU	tRNA	Amino acid	Codon	No.	RSCU	tRNA
Phe	UUU	999	1.33		Tyr	UAU	771	1.63	
Phe	UUC	499	0.67	*trnF-GAA*	Tyr	UAC	175	0.37	*trnY-GUA*
Leu	UUA	860	1.84	*trnL-UAA*	Stop	UAA	46	1.6	
Leu	UUG	569	1.22	*trnL-CAA*	Stop	UAG	22	0.77	
Leu	CUU	609	1.3		His	CAU	479	1.53	
Leu	CUC	180	0.38		His	CAC	146	0.47	*trnH-GUG*
Leu	CUA	394	0.84	*trnL-UAG*	Gln	CAA	723	1.54	*trnQ-UUG*
Leu	CUG	194	0.41		Gln	CAG	217	0.46	
Ile	AUU	1096	1.48		Asn	AAU	967	1.54	
Ile	AUC	461	0.62	*trnI-GAU*	Asn	AAC	289	0.46	*trnN-GUU*
Ile	AUA	667	0.9	*trnI-CAU*	Lys	AAA	1062	1.48	*trnK-UUU*
Met	AUG	629	1	*trn(f)M-CAU*	Lys	AAG	370	0.52	
Val	GUU	526	1.46		Asp	GAU	867	1.6	
Val	GUC	178	0.5	*trnV-GAC*	Asp	GAC	216	0.4	*trnD-GUC*
Val	GUA	542	1.51	*trnV-UAC*	Glu	GAA	1016	1.5	*trnE-UUC*
Val	GUG	191	0.53		Glu	GAG	343	0.5	
Ser	UCU	584	1.7		Cys	UGU	222	1.52	
Ser	UCC	344	1	*trnS-GGA*	Cys	UGC	70	0.48	*trnC-GCA*
Ser	UCA	398	1.16	*trnS-UGA*	Stop	UGA	18	0.63	
Ser	UCG	200	0.58		Trp	UGG	470	1	*trnW-CCA*
Pro	CCU	405	1.44		Arg	CGU	338	1.28	*trnR-ACG*
Pro	CCC	234	0.83		Arg	CGC	121	0.46	
Pro	CCA	324	1.15	*trnP-UGG*	Arg	CGA	353	1.33	
Pro	CCG	161	0.57		Arg	CGG	131	0.49	
Thr	ACU	539	1.63		Arg	AGA	488	1.84	*trnR-UCU*
Thr	ACC	247	0.75	*trnT-GGU*	Arg	AGG	159	0.6	
Thr	ACA	388	1.17	*trnT-UGU*	Ser	AGU	420	1.22	
Thr	ACG	150	0.45		Ser	AGC	115	0.33	*trnS-GCU*
Ala	GCU	603	1.73		Gly	GGU	539	1.21	
Ala	GCC	234	0.67		Gly	GGC	191	0.43	*trnG-GCC*
Ala	GCA	393	1.13	*trnA-UGC*	Gly	GGA	720	1.62	*trnG-UCC*
Ala	GCG	167	0.48		Gly	GGG	331	0.74	

RSCU: Relative Synonymous Codon Usage.

A given plant cell often contains multiple copies of cp genomes [51] that can be regarded as a population with genetic heterogeneity [5]. We mapped all SOLiD reads to the assembled genome to detect the possible polymorphic sites. However, no SNPs were recovered. A similar result was also observed in another cp genome, i.e., *Boea hygrome*trica (Gesneriaceae), which is also a member of the order Lamiales [46].

### Repeat Analysis

For repeat structure analysis, four forward, three inverted and seven tandem repeats were detected in the *Salvia miltiorrhiza* cp genome (Table 4). Most of these repeats exhibit lengths between 30 and 41 bp, while the CDS of the *ycf2* gene possesses the two longest tandem repeats at 63 and 108 bp. Three pairs of repeats associated with tRNA genes (Nos. 1, 5 and 6) and four tandem repeats (Nos. 8–11) in the intergenic spacers are distributed in the LSC region. A comparison of repeats between *Salvia miltiorrhiza* and *Sesamum indicum* shows that three repeats (Nos. 4, 5 and 7) are at the same location in the two cp genomes.

**Table 4:** Repeated sequences in the *Salvia miltiorrhiza* chloroplast genome.

Repeat number	Size (bp)	Type	Location	Repeat Unit	Region
1	30	F	*trnG-UCC*, *trnG-GCC*	AACGATGCGGGTTCGATTCCCGCTACCCGC	LSC
2	32	F	*psaB* (CDS), *psaA* (CDS)	AGCTAAATGATGATGAGCCATATCAGTCAACC	LSC
3	39	F	*ycf3* (intron), *ndhA* (intron)	CCAGAACCGTACGTGAGATTTTCACCTCATACGGCTCCT	LSC, SSC
4	41	F	IGS (*rps12*, *trnV*-*GAC*), *ndhA* (intron)	CTACAGAACCGTACATGAGATTTTCACCTCATACGGCTCCT	IRb, SSC
5	30	I	*trnS*-*GCU*, *trnS*-*GGA*	AACGGAAAGAGAGGGATTCGAACCCTCGGT	LSC
6	30	I	*trnS*-*UGA*, *trnS*-*GGA*	AGGGGAGAGAGAGGGATTCGAACCCTCGAT	LSC
7	41	I	*ndhA* (intron), IGS (*trnV*-*GAC*, rps12)	TTACAGAACCGTACATGAGATTTTCACCTCATACGGCTCCT	SSC, IRa
8	40	T	IGS (*rps16*, trnQ-UUG)	ACTATATAGAATATATATAA (×2)	LSC
9	32	T	IGS (*accD*, *psaI*)	TTAGCTTATCCGAATC (×2)	LSC
10	33	T	IGS (*accD*, *psaI*)	AATTAATAATAACTAC (×2)	LSC
11	34	T	IGS (*petA*, *psbJ*)	CGCACTCTTAGTCATAA (×2)	LSC
12	63	T	*ycf2* (CDS)	TTTTTGTCCAAGTCACTTCTT (×3)	IRb,a
13	108	T	*ycf2* (CDS)	TATTGATGAGAGTGACGA (×6)	IRb,a
14	39	T	*ndhF* (CDS)	AATAAAAACCTAAAATCCCT (×2)	SSC

F: Forward; I: Inverted; T: Tandem; IGS: Intergenic spacer; CDS: protein-coding regions. The underline represents the shared repeats with *Sesamum indicum*.

### SSR Analysis

SSRs, also known as microsatellites, are tandemly repeated DNA sequences that are generally 1–6 bp in length per unit and are distributed throughout the genome. SSRs have been accepted as one of the major sources of molecular markers due to their high polymorphism level within the same species and have been widely employed in population genetics and phylogenetic investigations [11], [52]–[54]. We detected perfect SSRs longer than 8 bp in *Salvia miltiorrhiza* together with 29 other asterid cp genomes. This threshold was set because SSRs of 8 bp or longer are prone to slip-strand mispairing, which is thought to be the primary mutational mechanism causing their high level of polymorphism [55]–[57]. In our analysis, the total number of SSRs ranged from 145 in *Panax ginseng* to 217 in *Anthriscus cerefolium* (Table 5), and a repertoire of 166 SSRs were detected in the *Salvia miltiorrhiza* cp genome. The majority of SSRs in all species are mononucleotides, varying in quantity from 92 in *Panax ginseng* to 155 in *Olea europaea.* Dinucleotides are the second most prevalent, ranging in quantity from 33 in *Helianthus annuus* to 62 in *Anthriscus cerefolium*. Generally, the number of tetranucleotides is slightly higher than that of trinucleotides, and only rarely are pentanucleotides or hexanucleotides observed in the asterid cp genomes. The majority of tri- to hexanucleotides are AT-rich in all species. An average of 68% (72% in *Salvia miltiorrhiza*) of all SSRs are A/T mononucleotides in these cp genomes, slightly lower than the 76% found in a previous study of 14 monocot cp genomes [56]. Our finding agrees with the contention that cp SSRs are generally composed of short polyadenine (polyA) or polythymine (polyT) repeats and rarely contain tandem guanine (G) or cytosine (C) repeats [58]. Thus, these SSRs contribute to the AT richness of the asterid cp genomes. We also detected SSRs in the CDS of each cp genome. The CDS accounts for approximately 50% of the total length in most cp genomes, whereas the SSR proportion ranges from 23% to 41%. This result indicates that SSRs are less abundant in CDS than in non-coding regions and that they are unevenly distributed within the cp genomes. In total, 53 SSRs were identified in the CDS of 23 genes in *Salvia miltiorrhiza*. Among them, 10 genes were found to harbor at least two SSRs, including *ndhD*, *matK*, *rpoC2*, *ycf1* and *ycf2*, among others.

**Table 5:** Distribution of SSRs present in the 30 asterid chloroplast genomes.

Taxon	Genome Size (bp)	AT (%)	SSR type							CDS		
			Mono	Di	Tri	Tetra	Penta	Hexa	Total	%^a^	No.^b^	%^c^
*Ageratina adenophora*	150,698	63	115	35	4	7	0	1	162	49	50	31
*Anthriscus cerefolium*	154,719	63	141	62	3	8	2	1	217	50	68	31
*Atropa belladonna*	156,687	62	117	46	2	9	0	0	174	51	50	29
*Boea hygrometrica*	153,493	62	98	40	3	8	0	0	149	52	46	31
*Coffea arabica*	155,189	63	115	46	3	4	0	0	168	51	57	34
*Datura stramonium*	155,871	62	109	40	3	8	0	0	160	52	53	33
*Daucus carota*	155,911	62	133	56	6	8	2	0	205	50	69	34
*Eleutherococcus senticosus*	156,768	62	109	47	2	7	0	0	165	50	56	34
*Guizotia abyssinica*	151,762	62	119	42	2	8	0	1	172	52	71	41
*Helianthus annuus*	151,104	62	119	33	4	4	0	0	160	51	63	39
*Ipomoea purpurea*	162,046	63	146	38	5	13	1	1	204	53	67	33
*Jacobaea vulgaris*	150,689	63	124	51	8	8	0	0	191	51	55	29
*Jasminum nudiflorum*	165,121	62	149	42	8	9	3	3	214	50	81	38
*Lactuca sativa*	152,765	62	118	49	3	2	0	0	172	48	43	25
*Nicotiana sylvestris*	155,941	62	118	41	5	9	1	0	174	54	62	36
*Nicotiana tabacum*	155,943	62	118	41	5	9	1	0	174	54	62	36
*Nicotiana tomentosiformis*	155,745	62	122	43	4	8	1	0	178	54	58	33
*Nicotiana undulata*	155,863	62	119	41	3	10	1	0	174	56	62	36
*Olea europaea*	155,888	62	155	35	0	4	2	0	196	51	50	26
*Olea europaea subsp. cuspidata*	155,862	62	152	36	0	3	2	0	193	51	46	24
*Olea europaea subsp. europaea*	155,875	62	153	35	0	3	2	0	193	51	46	24
*Olea europaea subsp. maroccana*	155,896	62	153	36	0	3	2	0	194	51	46	24
*Olea woodiana subsp. woodiana*	155,942	62	153	36	0	4	2	0	195	51	45	23
*Panax ginseng*	156,318	62	92	39	3	8	2	1	145	50	54	37
*Salvia miltiorrhiza*	151,328	62	122	35	0	8	0	1	166	52	53	32
*Sesamum indicum*	153,324	62	137	38	3	7	0	1	186	51	54	29
*Solanum bulbocastanum*	155,371	62	106	37	2	8	1	1	155	51	48	31
*Solanum lycopersicum*	155,461	62	114	33	1	7	1	0	156	51	46	29
*Solanum tuberosum*	155,296	62	103	36	2	8	1	0	150	51	46	31
*Trachelium caeruleum*	162,321	62	96	47	5	17	0	1	166	43	44	27

CDS: protein-coding regions.

a Percentage were calculated according to the total length of the CDS divided by the genome size.

b Total number of SSRs identified in the CDS.

c Percentage were calculated according to the total number of SSRs in the CDS divided by the total number of SSRs in the genome.

### Comparison with other cp Genomes in the Lamiales Order

Nine complete cp genome sequences of the Lamiales order are currently available, representing four families and five genera. Three sequences representing Gesneriaceae (*Boea hygrometrica*), Oleaceae (*Olea europaea*) and Pedaliaceae (*Sesamum indicum*) were selected for comparison with *Salvia miltiorrhiza*. *Epifagus virginiana* (Orobanchaceae) was not considered because most cp genes are lost in this non-green parasitic flowering plant [7]. *Jasminum nudiflorum* (Oleaceae) was also excluded due to its genome rearrangements [8].

The genome size of *Salvia miltiorrhiza* is the smallest of the Lamiales cp genomes, with the exception of *Epifagus virginiana*. It is approximately 2.2 kb, 4.6 kb and 2.0 kb smaller than that of *Boea hygrometrica*, *Olea europaea* and *Sesamum indicum*, respectively. This variation in sequence length is mainly attributed to the difference in the length of the LSC region (Table S4).

**Figure 2:**
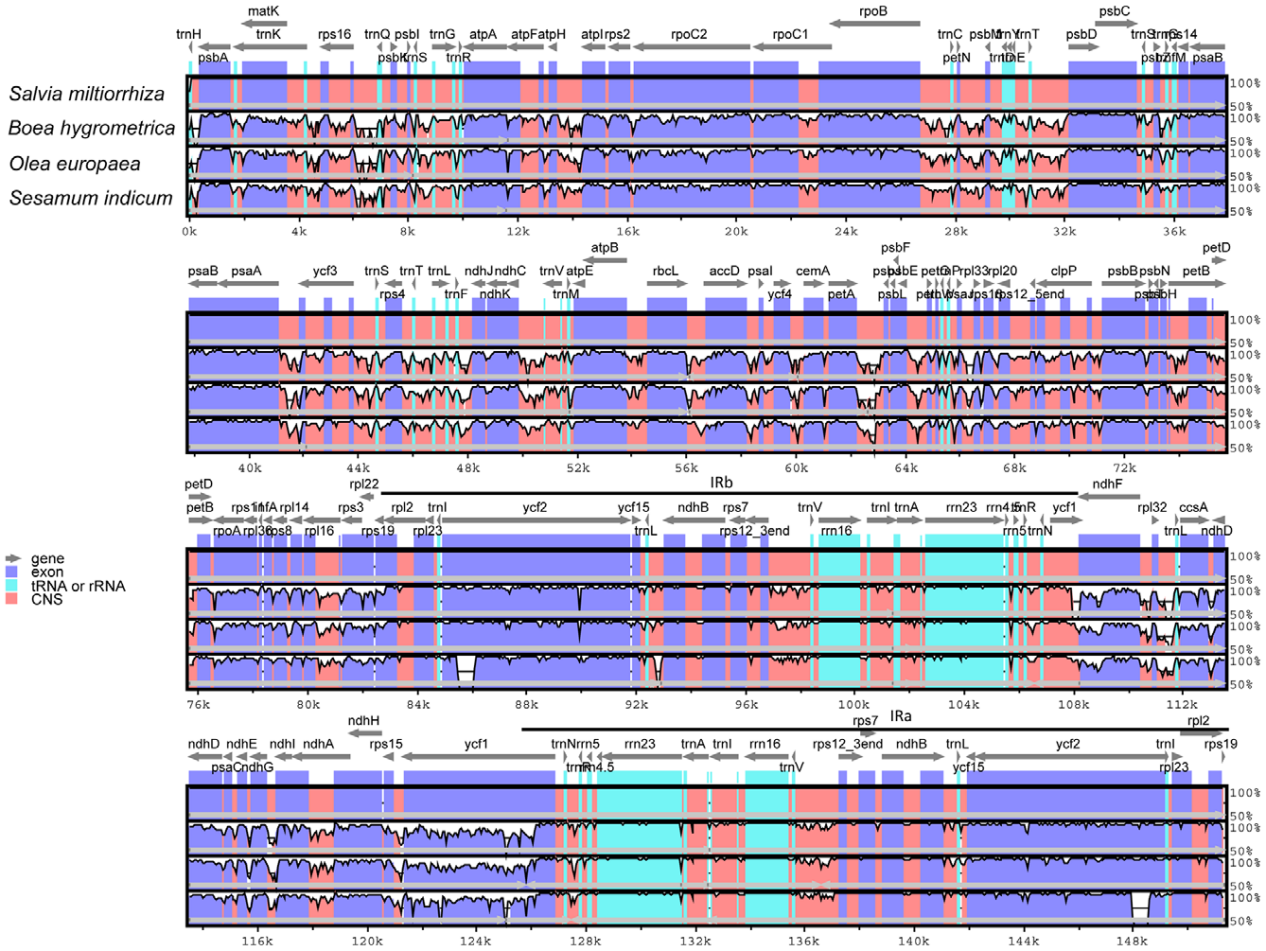
Comparison of four chloroplast genome using mVISTA program. Grey arrows and thick black lines above the alignment indicate genes with their orientation and the position of the IRs, respectively. A cut-off of 70% identity was used for the plots, and the Y-scale represents the percent identity between 50–100%. Genome regions are color-coded as protein-coding (exon), rRNA, tRNA and conserved noncoding sequences (CNS).

Pairwise cp genomic alignment between *Salvia miltiorrhiza* and the three cp genomes recovered a high degree of synteny (Figure S1, S2, S3). Since the cp genome of tobacco is often regarded to be unarranged [48], we compared the four cp genomes with it and observed an approximately identical gene order and organization among them. The overall sequence identity of the four Lamiales cp genomes was plotted using mVISTA using the annotation of *Salvia miltiorrhiza* as reference (Figure 2). The comparison shows that the two IR regions are less divergent than the LSC and SSC regions. Additionally, non-coding regions exhibit a higher divergence than coding regions, and the most divergent regions localize in the intergenic spacers among the four cp genomes. In our alignment, these highly divergent regions include *ndhD*-*ccsA*, *ndhI*-*ndhG*, *psbI*-*trnS* and *trnH*-*psbA,* among others. Similar results were also observed in the non-coding region comparison of six Asteraceae cp genomes [36]. Cp non-coding regions have been successfully applied in phylogenetic analysis of Lamiales [59], [60] and in the DNA barcoding research presented in a growing number of studies [61], [62]. Variation between the coding sequences of *Salvia miltiorrhiza* and *Boea hygrometrica*, *Olea europaea* or *Sesamum indicum* was also analyzed by comparing each individual gene as well as the overall sequences (Table S5) [63]. The four rRNA genes are the most conserved, while the most divergent coding regions are *rpl22*, *ycf1*, *ndhF*, *ccsA*, *rps15* and *matK*.

### IR Contraction and Expansion

**Figure 3:**
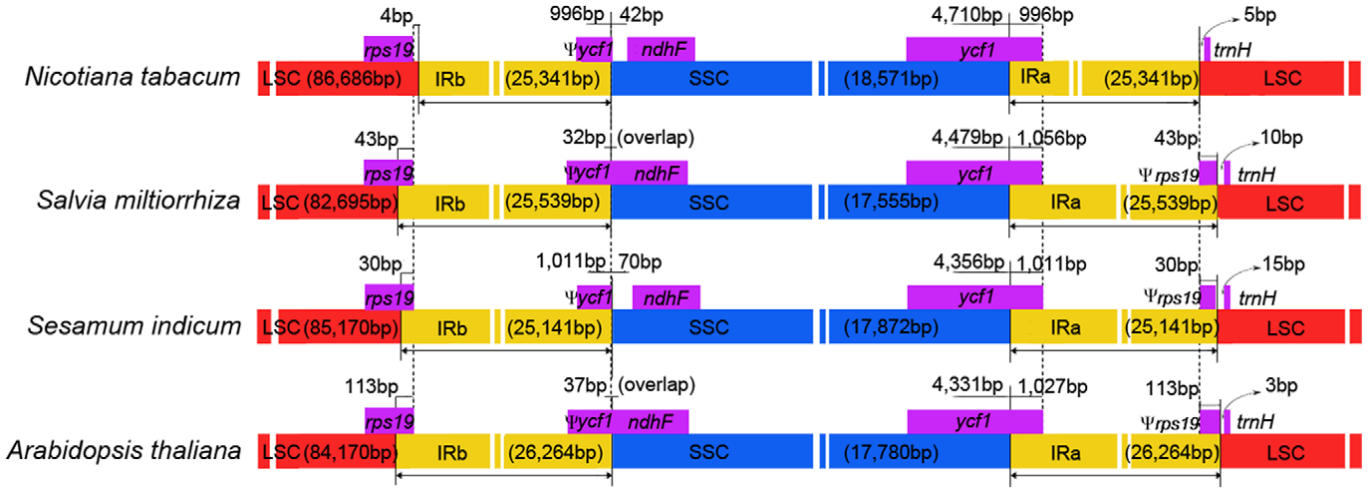
Comparison of the borders of LSC, SSC and IR regions among four chloroplast genomes. The IRb/SSC border extended into the *ycf1* genes to create various lengths of *ycf1* pseudogenes among four chloroplast genomes. The *ycf1* pseudogene and the *ndhF* gene overlapped in both the *Salvia miltiorrhiza* and *Arabidopsis thaliana* cp genomes by 32 bp and 37 bp, respectively. Various lengths of *rps19* pseudogenes were created at the IRa/LSC borders of *Salvia miltiorrhiza*, *Sesamum indicum* and *Arabidopsis thaliana*. This figure is not to scale.

Although IRs are the most conserved regions in the cp genomes, the contraction and expansion at the borders of the IR regions are common evolutionary events and represent the main reasons for size variation of cp genomes [5], [57], [64], [65]. The IR-LSC and IR-SSC borders of the cp genomes of *Arabidopsis thaliana*, *Nicotiana tabacum*, *Sesamum indicum*, *Salvia miltiorrhiza* were compared, and those data are presented in Figure 3.

The IRb/SSC border extended into the *ycf1* genes to create long *ycf1* pseudogenes in all of the species compared. The length of *ycf1* pseudogene was 996 bp in *Nicotiana tabacum*, 1,011 bp in *Sesamum indicum*, 1,056 bp in *Salvia miltiorrhiza* and 1,027 bp in *Arabidopsis thaliana*. In addition, the *ycf1* pseudogene and the *ndhF* gene overlapped in both the *Salvia miltiorrhiza* and *Arabidopsis thaliana* cp genomes by 32 bp and 37 bp, respectively. The IRa/SSC border was located in the CDS of *ycf1* gene and expanded the same length into the 5′ portion of *ycf1* gene as IRb expanded in the four cp genomes. *Rps19* pseudogenes of various lengths were also found at the IRa/LSC borders. In *Salvia miltiorrhiza*, a short *rps19* pseudogene of 43 bp was created at the IRa/LSC border. The same pseudogene was 30 bp and 113 bp in *Sesamum indicum* and *Arabidopsis thaliana*, respectively, and was not found at the same border of *Nicotiana tabacum*. The *trnH* genes of these four species were all located in the LSC region, 3–15 bp apart from the IRa/LSC border, whereas this gene was usually located in the IR region in the monocot cp genomes [56].

### Sequence Divergence of Protein-coding Genes

We compared gene contents and calculated the average pairwise sequence distance of 80 protein-coding genes among 30 asterid species. The abnormal or missing annotations of several genes in some taxa were re-annotated during the sequence analysis. The results are summarized in Table S6.

Low levels of average sequence distance among the asterid coding sequences were observed. 85% of these genes have an average sequence distance less than 0.10, and only 12 genes exhibit an average sequence distance greater than 0.10. The ten most divergent genes are *ycf15*, *ycf1*, *rpl22*, *rpl32*, *matK*, *clpP*, *ndhF*, *ccsA*, *rps15* and *accD*. The highest average sequence distance was observed in *ycf15* (0.41), followed by *ycf1* (0.28). The latter is located at the LSC/IR border and shows a fast evolving trend. Previously reported comparison of each individual region revealed different sets of the most divergent genes in the different cp genomes analyzed. *RpoC1* and *ycf1* were identified to be the most divergent genes in six Asteraceae cp genomes [36]; *ycf1*, *accD*, *clpP*, *rps16* and *ndhA* were observed to be the most divergent coding regions in *Parthenium argentatum* and its closely related species [63]; *ycf1*, *matK*, *accD*, *rpl22*, *infA*, *ycf2*, *rps15*, *ccsA* and *rpl32* were the most divergent genes in 16 vascular plant cp genomes [47]. The most divergent genes in asterids are similar for most of the genes indicated above, but they also include *ndhF* and *ycf15*.

The ten most conserved genes are *ndhB*, *rpl2*, *psbL*, *petG*, *rps7*, *rpl23*, *psbN*, *psbF*, *psbZ* and *psbA*. Of them, the three *rpl* and *rps* genes located in IR regions show lower average sequence distances than the other *rpl* or *rps* genes located in the LSC or SSC regions. This supports the hypothesis that sequences in the IR regions diverge at a slower rate than sequences located in the LSC or SSC regions. This slower divergence may occur because the two IR regions suffer frequent intra-molecular recombination events, which provide selective constraints on both sequence homogeneity and structural stability [44]. However, some genes (e.g. *ycf2* and the 3′ end of *rps12*) in IRs exhibit more variation than several genes in the LSC or SSC regions. Furthermore, the *ycf15* gene was found to be 31 times more diverse than the *nhdB* gene, though both genes are located in the IRs. In addition to the effect of regional constraints on sequence evolution, functional constraints were also demonstrated to affect the divergence levels of genes in asterids. For example, the majority of the *psa*, *psb* and *pet* gene classes show relatively slow evolutionary divergence. Similar results were also observed in the study of Kim and Lee [47].

The gene contents are relatively conserved among the 30 asterid cp genomes, with the exception of some species. The *accD* gene becomes pseudogene in *Jasminum nudiflorum* and *Trachelium caeruleum*. In addition to *accD,* the five genes *clpP*, *infA*, *ndhK*, *rpl23* and *ycf15* exist as pseudogenes in *Trachelium caeruleum*. *PsbI* and *rps19* exist as pseudogenes in *Boea hygrometrica*. *InfA* and *ycf15* were lost in 10 and 17 species, respectively. In terms of length variation, 14 genes show no variation, and 20 genes show less than 10 bp variation. The majority of these length-conserved genes belong to the *psa*, *psb* and *pet* gene classes. In addition, large-scale sequence length variation (>1,000 bp) was observed in *ycf1* and *ycf2*. The length variation of *ycf1* is attributed to the indel mutation and IR contraction and expansion, and the length variation of *ycf2* is caused by the internal indel mutation associated with short direct repeats [47], [66]. When both sequence divergence and length variation are considered, *ycf1* and *ycf2*, together with *accD*, *clpP*, *ndhF* and *matK*, are probably good candidates for phylogenetic studies among closely related species in asterids.

### Phylogenetic Analysis

To identify the phylogenetic position of *Salvia miltiorrhiza* within the asterid lineage, we performed multiple sequence alignments using 71 protein-coding genes commonly present in the aforementioned cp genomes. The 30 complete cp genomes represent 10 families within five orders of asterids, including Apiaceae, Araliaceae, Asteraceae, Convolvulaceae, Gesneriaceae, Lamiaceae, Oleaceae, Pedaliaceae, Rubiaceae and Solanaceae (Table S2). Two additional eudicot cp genomes, *Spinacia oleracea* and *Arabidopsis thaliana,* were set as outgroups. The sequence alignment data matrix used for phylogenetic analysis comprised 62,939 nucleotide positions, which was reduced to 54,400 characters when gaps were excluded to avoid alignment ambiguities due to length variation.

**Figure 4:**
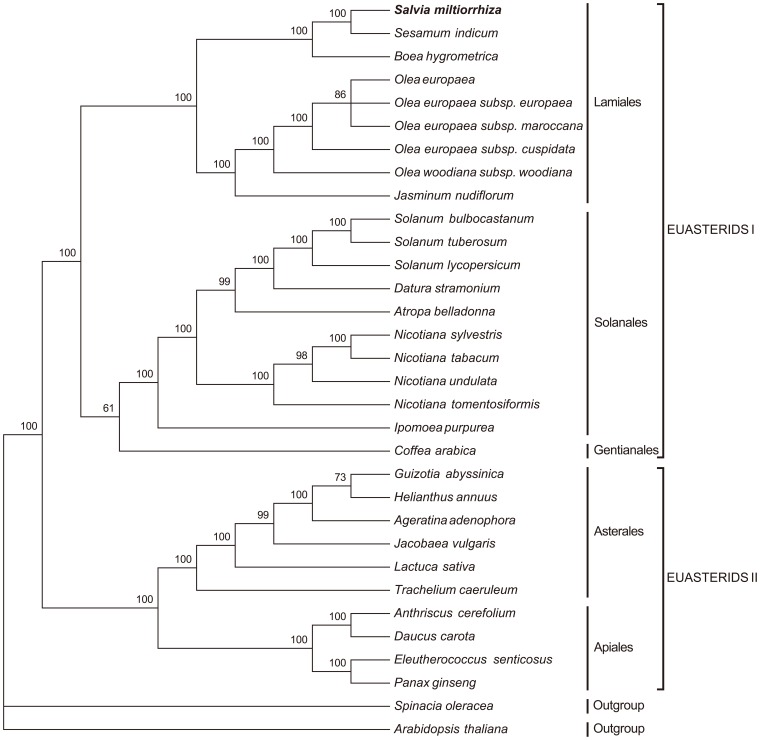
The MP phylogenetic tree of the asterid clade based on 71 protein-coding genes. The MP tree has a length of 36,088, with a consistency index of 0.6628 and a retention index of 0.7561. Numbers above each node are bootstrap support values. *Spinacia oleracea* and *Arabidopsis thaliana* were set as outgroups.

MP analysis resulted in a single tree with a length of 36,088, a consistency index of 0.6628 and a retention index of 0.7561 (Figure 4). Bootstrap analysis showed that there were 25 out of 28 nodes with bootstrap values >95%, and 22 of these had a bootstrap value of 100%. A ML tree was obtained with the -lnL of 264933.3750 using the GTR+I+G nucleotide substitution model (Figure S4). ML bootstrap values were also high, with values of >95% for 25 of the 28 nodes, and 24 nodes with 100% bootstrap support. Both MP and ML trees had similar phylogenetic topologies, which formed two major clades, euasterids I and II. The only incongruence between the MP and ML trees was the position of *Coffea*. In the MP tree, *Coffea* was placed sister to Solanales; whereas it was positioned close to Lamiales in the ML tree. Bootstrap supporting values (61% in MP and 65% in ML) for these alternative placements were weak. Both the MP and ML phylogenetic results strongly supported, with 100% bootstrap values, the position of *Salvia miltiorrhiza* as the sister of the closely related species *Sesamum indicum* in the order Lamiales.

## Discussion

### Genome Organization

The *Salvia miltiorrhiza* cp genome with a pair of IRs separating the LSC and SSC regions exhibits identical gene order and content to most sequenced angiosperm cp genomes, emphasizing the highly conserved nature of these land plant cp genomes [2]. Repeat analysis revealed four forward, three inverted and seven tandem repeats in the *Salvia miltiorrhiza* cp genome. Most of these repeats are located in the intergenic spacers and introns, but several occur in tRNAs and CDS. Short dispersed repeats are considered to be one of the major factors promoting cp genome rearrangements because they are common in highly rearranged algal and angiosperm genomes, and many rearrangement endpoints are associated with such repeats [8], 67–70. The role of short dispersed repeats in unrearranged cp genomes is still unclear [71], [72]. All of these repeats, together with the aforementioned SSRs, are informative sources for developing markers for population studies [36].

### Phylogenetic Relationships

Chloroplast genomes provide rich sources of phylogenetic information, and numerous studies using cp DNA sequences have been carried out during the past two decades, greatly enhancing our understanding of the evolutionary relationships among angiosperms [9], [73], [74]. *Salvia*, consisting of nearly 1,000 species, is the largest genus in the Lamiaceae family and is widely distributed throughout the world [75]. Previous phylogenetic studies employing one or several genes or intergenic regions showed evidence of a polyphyletic nature of *Salvia* [75], [76]. Our phylogenies based on 71 protein-coding genes placed *Salvia* sister to *Sesamum* in asterids with strong support and resolution. Both trees are congruent to that in a recent study using 32 complete asterid cp genomes [44] and to the APG tree [25]. The incongruence between the MP and ML trees regarding the position of *Coffea* is likely due to the limited number of complete cp genomes in Gentianales. Thus, to acquire more accurate relationships in asterids, expanded taxon sampling will be required for this large and diverse clade of angiosperms.

### Implications for Chloroplast Genetic Engineering

Chloroplast genetic engineering is exemplary for its unique advantages including the possibility of multi-gene engineering in a single transformation event, transgene containment due to maternal inheritance, high levels of transgene expression and lack of gene silencing [77]–[79]. Significant progress in chloroplast transformation has been made in the model species tobacco as well as in a few major crops [78], [79]. Although the *trnI*/*trnA* and *accD*/*rbcL* intergenic spacer regions have been widely used as gene introduction sites for vector construction [79], the transformation efficiency is impaired when the sequences for homologous recombination are divergent among distantly related species [71]. The availability of the complete cp genome sequence of *Salvia miltiorrhiza* is helpful to identify the optimal intergenic spacers for transgene integration and to develop site-specific cp transformation vectors. The genes related to its bioactive compound synthesis [21], [24] will be the primary targets for investigation in *Salvia*. In addition, using cp genetic engineering to introduce useful traits, such as herbicide resistance and drought tolerance, might be other applications to improve this medicinal plant.

### Conclusion

We present the first complete cp genome from Lamiaceae family using both pyrosequencing and SOLiD technologies. The gene order and genome organization of *Salvia miltiorrhiza* cp sequence are similar to that of tobacco and three other cp genomes in the Lamiales. Further, the distribution and location of repeated sequences were determined. SSR, protein-coding gene sequence divergence and phylogenetic analysis were performed among 30 asterid cp genomes. All the data presented in this paper will facilitate the biological study of this important medicinal plant.

## Supporting Information

Figure S1 **Chloroplast genomic alignment between** ***Salvia miltiorrhiza*** **and** ***Boea hygrometrica*** **.**
(TIF)

Figure S2 **Chloroplast genomic alignment between** ***Salvia miltiorrhiza*** **and** ***Olea europaea*** **.**
(TIF)

Figure S3 **Chloroplast genomic alignment between** ***Salvia miltiorrhiza*** **and** ***Sesamum indicum*** **.**
(TIF)

Figure S4 **The ML phylogenetic tree (−lnL = 264933.3750) of the asterid clade based on 71 protein-coding genes.** The GTR+I+G nucleotide substitution model was adopted based on the Modeltest. Numbers above each node are bootstrap support values. *Spinacia oleracea* and *Arabidopsis thaliana* were set as outgroups.
(TIF)

Table S1 **Primers used for assembly validation.**
(DOC)

Table S2 **The list of accession numbers of the chloroplast genome sequences used in this study.**
(DOC)

Table S3 **Genes present in the** ***Salvia miltiorrhiza*** **chloroplast genome.**
(DOC)

Table S4 **Size comparison of** ***Salvia miltiorrhiza*** **chloroplast genomic regions with three other Lamiales chloroplast genomes.**
(DOC)

Table S5 **Comparison of homologues between the** ***Salvia miltiorrhiza*** **and** ***Boea hygrometrica*** **(** ***Bh*** **),** ***Olea europaea*** **(** ***Oe*** **) or** ***Sesamum indicum*** **(** ***Si*** **) chloroplast genomes using the percent identity of protein-coding sequences.**
(DOC)

Table S6 **Average pairwise sequence distance of protein-coding genes among the 30 asterid chloroplast genomes.**
(DOC)
