# Supplementary figures and images for: The Complete Chloroplast Genome Sequence of the Medicinal Plant Salvia miltiorrhiza
Source: PLoS One. 2013 Feb 27;8(2):e57607. doi: 10.1371/journal.pone.0057607 (PMC3584094; doi:10.1371/journal.pone.0057607)

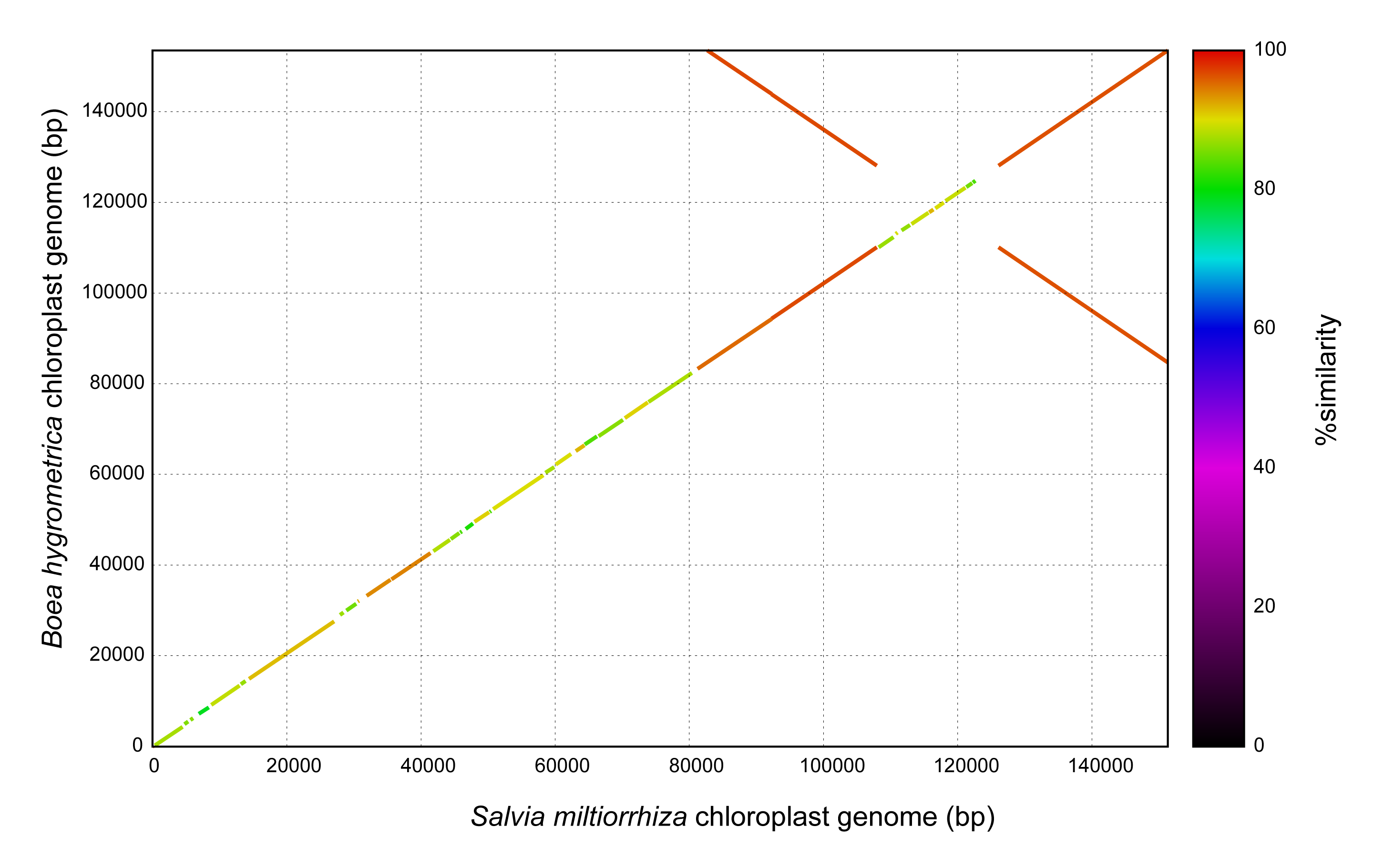

Supplement: Figure S1 — Chloroplast genomic alignment between Salvia miltiorrhiza and Boea hygrometrica . (TIF) [file pone.0057607.s001.tif]

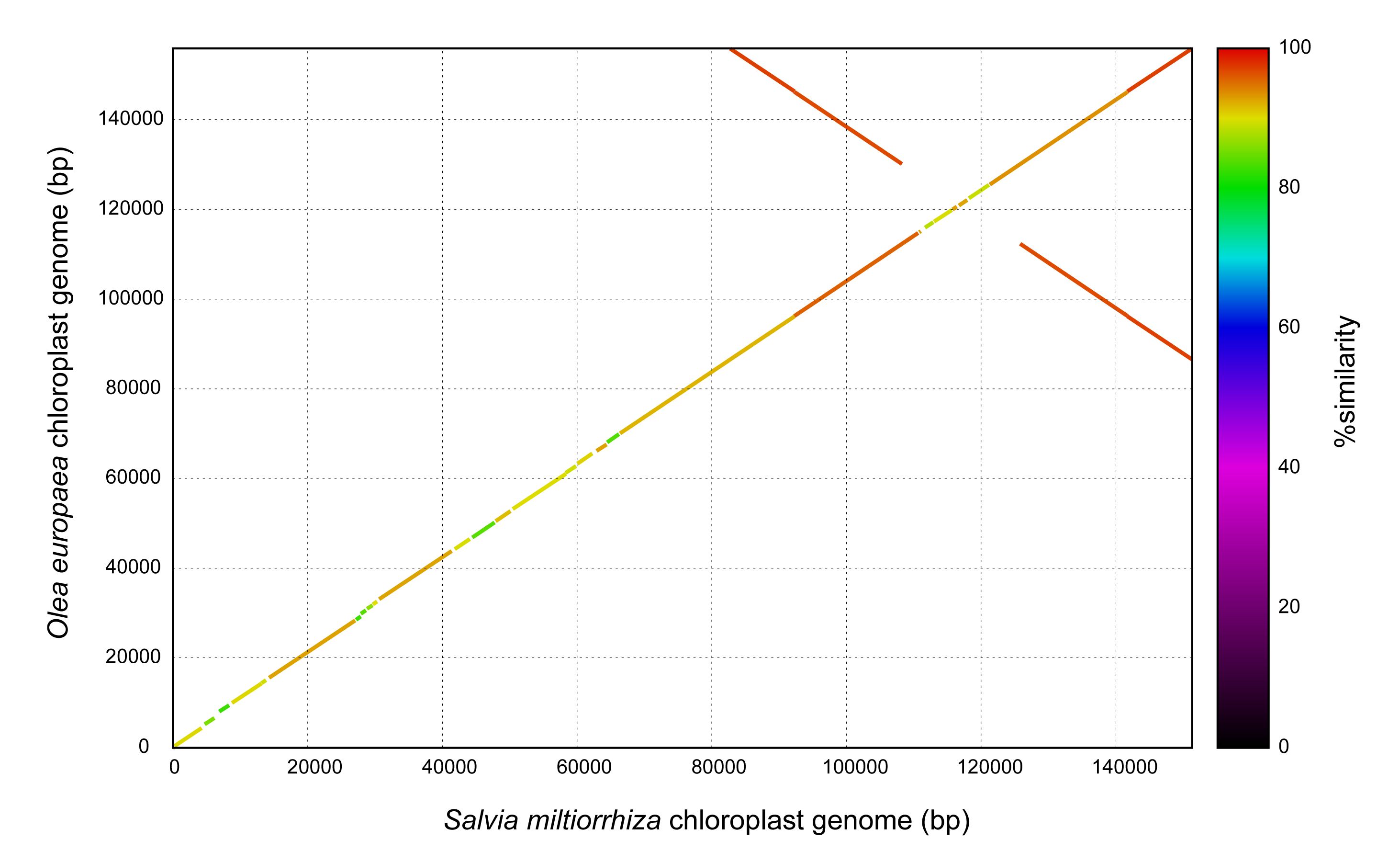

Supplement: Figure S2 — Chloroplast genomic alignment between Salvia miltiorrhiza and Olea europaea . (TIF) [file pone.0057607.s002.tif]

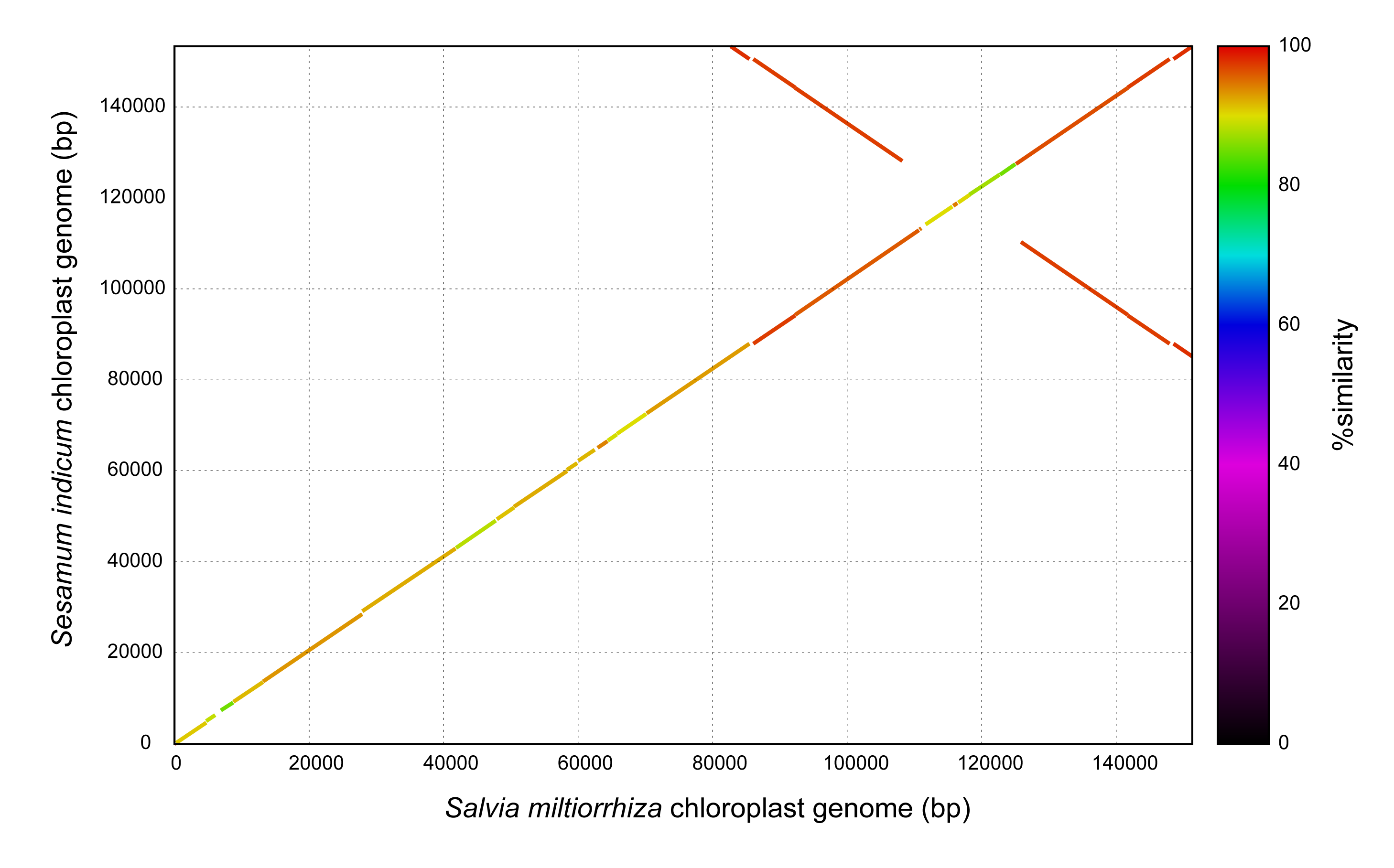

Supplement: Figure S3 — Chloroplast genomic alignment between Salvia miltiorrhiza and Sesamum indicum . (TIF) [file pone.0057607.s003.tif]

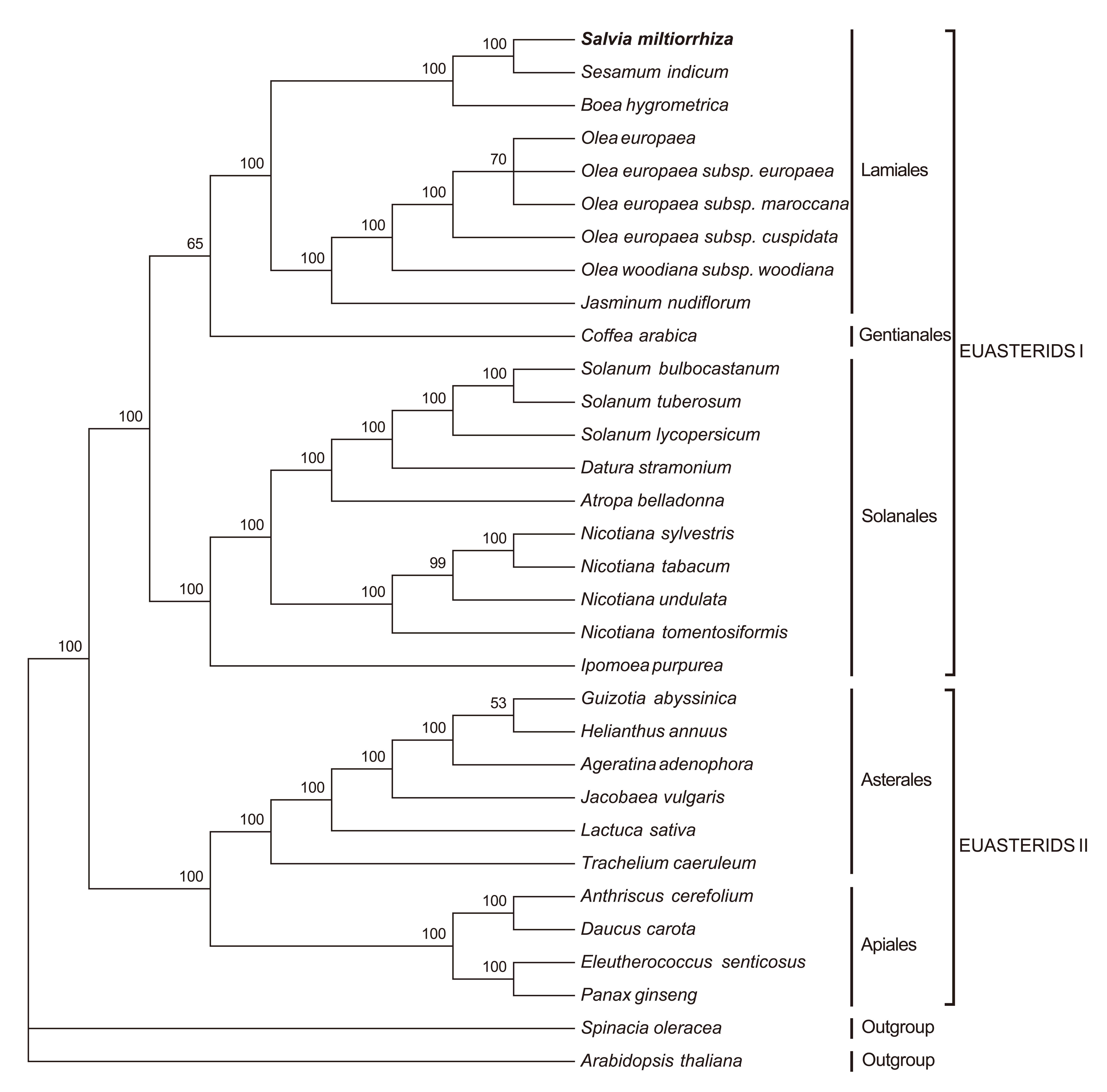

Supplement: Figure S4 — The ML phylogenetic tree (−lnL = 264933.3750) of the asterid clade based on 71 protein-coding genes. The GTR+I+G nucleotide substitution model was adopted based on the Modeltest. Numbers above each node are bootstrap support values. Spinacia oleracea and Arabidopsis thaliana were set as outgroups. (TIF) [file pone.0057607.s004.tif]
