# Supplementary material for: The Complete Chloroplast Genome Sequence of the Medicinal Plant Salvia miltiorrhiza
Source: PLoS One. 2013 Feb 27;8(2):e57607. doi: 10.1371/journal.pone.0057607 (PMC3584094; doi:10.1371/journal.pone.0057607)
Supplement: Table S1 — Primers used for assembly validation. (DOC) [file pone.0057607.s005.doc]

**Table S1.** Primers used for assembly validation.

| Primer | Sequence (5’>3’) | Amplicon size (bp) |
| --- | --- | --- |
| 1 (IRa/LSC) | F CCAGAAAAGTTTGGGTAGAGCCG | 819 |
|  | R CAACCGTGCTAACCTTGGTATGG |  |
| 2 (LSC/IRb) | F ACTAGCATTTGCTGCGGCAG | 915 |
|  | R AAAATTACCTTCTGGGGAGGTCC |  |
| 3 (IRb/SSC) | F GGCTTGGGTTGGTATTAGTCTGG | 689 |
|  | R CTGGGGGAATAAGAGAATTGGC |  |
| 4 (SSC/IRa) | F GGATCTTTCAGGTTTAGACTGGGAG | 865 |
|  | R CGGAAGAAATCCGAGTGAATGG |  |
