# Supplementary material for: The Complete Chloroplast Genome Sequence of the Medicinal Plant Salvia miltiorrhiza
Source: PLoS One. 2013 Feb 27;8(2):e57607. doi: 10.1371/journal.pone.0057607 (PMC3584094; doi:10.1371/journal.pone.0057607)
Supplement: Table S2 — The list of accession numbers of the chloroplast genome sequences used in this study. (DOC) [file pone.0057607.s006.doc]

**Table S2.** The list of accession numbers of the chloroplast genome sequences used in this study.

| No. | Taxon | Family | Order | GenBank Accession number |
| --- | --- | --- | --- | --- |
| 1 | *Salvia miltiorrhiza* | Lamiaceae | Lamiales | JX312195 |
| 2 | *Ageratina adenophora* | Asteraceae | Asterales | NC_015621 |
| 3 | *Anthriscus cerefolium* | Apiaceae | Apiales | NC_015113 |
| 4 | *Daucus carota* | Apiaceae | Apiales | NC_008325 |
| 5 | *Eleutherococcus senticosus* | Araliaceae | Apiales | NC_016430 |
| 6 | *Guizotia abyssinica* | Asteraceae | Asterales | NC_010601 |
| 7 | *Helianthus annuus* | Asteraceae | Asterales | NC_007977 |
| 8 | *Jacobaea vulgaris* | Asteraceae | Asterales | NC_015543 |
| 9 | *Lactuca sativa* | Asteraceae | Asterales | NC_007578 |
| 10 | *Panax ginseng* | Araliaceae | Apiales | NC_006290 |
| 11 | *Trachelium caeruleum* | Asteraceae | Asterales | NC_010442 |
| 12 | *Atropa belladonna* | Solanaceae | Solanales | NC_004561 |
| 13 | *Boea hygrometrica* | Gesneriaceae | Lamiales | NC_016468 |
| 14 | *Coffea arabica* | Rubiaceae | Gentianales | NC_008535 |
| 15 | *Datura stramonium* | Solanaceae | Solanales | NC_018117 |
| 16 | *Ipomoea purpurea* | Convolvulaceae | Solanales | NC_009808 |
| 17 | *Jasminum nudiflorum* | Oleaceae | Lamiales | NC_008407 |
| 18 | *Nicotiana sylvestris* | Solanaceae | Solanales | NC_007500 |
| 19 | *Nicotiana tabacum* | Solanaceae | Solanales | NC_001879 |
| 20 | *Nicotiana tomentosiformis* | Solanaceae | Solanales | NC_007602 |
| 21 | *Nicotiana undulata* | Solanaceae | Solanales | NC_016068 |
| 22 | *Olea europaea* | Oleaceae | Lamiales | NC_013707 |
| 23 | *Olea europaea subsp. cuspidata* | Oleaceae | Lamiales | NC_015604 |
| 24 | *Olea europaea subsp. europaea* | Oleaceae | Lamiales | NC_015401 |
| 25 | *Olea europaea subsp. maroccana* | Oleaceae | Lamiales | NC_015623 |
| 26 | *Olea woodiana subsp. woodiana* | Oleaceae | Lamiales | NC_015608 |
| 27 | *Sesamum indicum* | Pedaliaceae | Lamiales | NC_016433 |
| 28 | *Solanum bulbocastanum* | Solanaceae | Solanales | NC_007943 |
| 29 | *Solanum lycopersicum* | Solanaceae | Solanales | NC_007898 |
| 30 | *Solanum tuberosum* | Solanaceae | Solanales | NC_008096 |
| 31 | *Spinacia oleracea* | Amaranthaceae | Caryophyllales | NC_002202 |
| 32 | *Arabidopsis thaliana* | Brassicaceae | Brassicales | NC_000932 |
