# Supplementary material for: The Complete Chloroplast Genome Sequence of the Medicinal Plant Salvia miltiorrhiza
Source: PLoS One. 2013 Feb 27;8(2):e57607. doi: 10.1371/journal.pone.0057607 (PMC3584094; doi:10.1371/journal.pone.0057607)
Supplement: Table S4 — Size comparison of Salvia miltiorrhiza chloroplast genomic regions with three other Lamiales chloroplast genomes. (DOC) [file pone.0057607.s008.doc]

**Table S4. Size comparison of *Salvia miltiorrhiza* chloroplast genomic regions with three other Lamiales chloroplast genomes.**

| Species | Length (bp) | | | |
| --- | --- | --- | --- | --- |
|  | Total genome | LSC | SSC | IR |
| *Salvia miltiorrhiza* | 151,328 | 82,695 | 17,555 | 25,539 |
| *Boea hygrometrica* | 153,493 | 84,692 | 17,901 | 25,450 |
| *Olea europaea* | 155,888 | 86,590 | 17,816 | 25,741 |
| *Sesamum indicum* | 153,324 | 85,170 | 17,872 | 25,141 |
