# Supplementary material for: The Complete Chloroplast Genome Sequence of the Medicinal Plant Salvia miltiorrhiza
Source: PLoS One. 2013 Feb 27;8(2):e57607. doi: 10.1371/journal.pone.0057607 (PMC3584094; doi:10.1371/journal.pone.0057607)
Supplement: Table S5 — Comparison of homologues between the Salvia miltiorrhiza and Boea hygrometrica ( Bh ), Olea europaea ( Oe ) or Sesamum indicum ( Si ) chloroplast genomes using the percent identity of protein-coding sequences. (DOC) [file pone.0057607.s009.doc]

**Table S5. Comparison of homologues between the *Salvia miltiorrhiza* and *Boea hygrometrica* (*Bh*), *Olea* *europaea* (*Oe*) or *Sesamum* *indicum* (*Si*) chloroplast genomes using the percent identity of protein-coding sequences.**

| Gene | Start | End | Length (bp) | % identity | | |
| --- | --- | --- | --- | --- | --- | --- |
|  |  |  |  | *Bh* | *Oe* | *Si* |
| *accD* | 56642 | 58190 | 1488 | 91.97 | 92.11 | 93.04 |
| *atpA* | 10038 | 11561 | 1524 | 93.83 | 94.95 | 95.67 |
| *atpB* | 52298 | 53794 | 1497 | 95.52 | 95.79 | 96.26 |
| *atpE* | 51900 | 52301 | 402 | 92.27 | 96.13 | 96.27 |
| *atpF* | 11656 | 12909 | 555 | 83.41 | 95.68 | 96.22 |
| *atpH* | 13183 | 13428 | 246 | 95.53 | 95.93 | 97.97 |
| *atpI* | 14399 | 15142 | 744 | 94.89 | 96.10 | 96.91 |
| *ccsA* | 111902 | 112879 | 978 | 87.42 | 89.24 | 90.43 |
| *cemA* | 60320 | 61009 | 690 | 92.21 | 93.88 | 95.51 |
| *clpP* | 68855 | 70765 | 591 | 91.41 | 95.94 | 94.75 |
| *infA* | 78406 | 78639 | 234 | 93.59 | 94.17 | 94.87 |
| *matK* | 1972 | 3534 | 1563 | 87.36 | 91.81 | 91.64 |
| *ndhA* | 117272 | 119348 | 1092 | 90.81 | 92.13 | 93.77 |
| *ndhB* | 92999  138818 | 95206  141025 | 1533 | 98.89 | 99.48 | 99.61 |
| *ndhC* | 49483 | 49845 | 363 | 95.04 | 97.52 | 97.52 |
| *ndhD* | 113123 | 114652 | 1530 | 91.78 | 92.48 | 93.21 |
| *ndhE* | 115258 | 115563 | 306 | 94.77 | 94.77 | 97.06 |
| *ndhF* | 108203 | 110419 | 2217 | 83.58 | 88.47 | 89.36 |
| *ndhG* | 115779 | 116309 | 531 | 93.22 | 93.97 | 95.29 |
| *ndhH* | 119350 | 120531 | 1182 | 94.33 | 94.08 | 95.35 |
| *ndhI* | 116685 | 117191 | 507 | 92.50 | 95.72 | 94.48 |
| *ndhJ* | 48167 | 48643 | 477 | 93.92 | 96.44 | 97.06 |
| *ndhK* | 48754 | 49431 | 678 | 93.66 | 93.95 | 95.70 |
| *petA* | 61215 | 62177 | 963 | 94.70 | 95.53 | 96.26 |
| *petB* | 73670 | 76451 | 651 | 96.52 | 96.92 | 97.67 |
| *petD* | 75213 | 76415 | 483 | 94.96 | 97.72 | 97.52 |
| *petG* | 65096 | 65209 | 114 | 98.25 | 100.00 | 100.00 |
| *petL* | 64822 | 64917 | 96 | 96.88 | 95.74 | 98.96 |
| *petN* | 28062 | 28151 | 90 | 98.84 | 98.89 | 100.00 |
| *psaA* | 38839 | 41091 | 2253 | 96.40 | 96.85 | 98.05 |
| *psaB* | 36609 | 38813 | 2205 | 95.46 | 95.96 | 97.10 |
| *psaC* | 114762 | 115007 | 246 | 92.28 | 95.12 | 94.31 |
| *psaI* | 58653 | 58763 | 111 | 95.50 | 96.40 | 98.20 |
| *psaJ* | 65932 | 66066 | 135 | 98.47 | 97.71 | 99.24 |
| *psbA* | 392 | 1450 | 1059 | 96.49 | 97.33 | 98.10 |
| *psbB* | 71214 | 72740 | 1527 | 95.42 | 95.87 | 96.79 |
| *psbC* | 33163 | 34584 | 1422 | 96.34 | 96.27 | 97.05 |
| *psbD* | 32154 | 33215 | 1062 | 98.31 | 97.16 | 98.68 |
| *psbE* | 63764 | 64015 | 252 | 96.03 | 98.41 | 98.81 |
| *psbF* | 63630 | 63749 | 120 | 96.67 | 98.33 | 97.50 |
| *psbH* | 73323 | 73544 | 222 | 95.50 | 99.10 | 97.30 |
| *psbI* | 7953 | 8063 | 111 | N/A | 97.30 | 100.00 |
| *psbJ* | 63238 | 63360 | 123 | 97.56 | 95.93 | 97.56 |
| *psbK* | 7377 | 7556 | 180 | 95.38 | 92.39 | 92.93 |
| *psbL* | 63490 | 63606 | 117 | 95.73 | 97.44 | 97.44 |
| *psbM* | 29128 | 29232 | 105 | 95.24 | 98.10 | 97.14 |
| *psbN* | 73086 | 73217 | 132 | 96.95 | 96.97 | 98.48 |
| *psbT* | 72918 | 73025 | 108 | 95.96 | 93.52 | 95.37 |
| *psbZ* | 35257 | 35445 | 189 | 97.35 | 97.88 | 98.41 |
| *rbcL* | 54579 | 56012 | 1434 | 95.24 | 95.92 | 96.50 |
| *rpl14* | 79360 | 79728 | 369 | 95.39 | 96.75 | 97.56 |
| *rpl16* | 79860 | 81140 | 408 | 91.67 | 94.74 | 96.57 |
| *rpl2* | 82798  149744 | 84280  151226 | 825 | 98.68 | 99.15 | 99.52 |
| *rpl20* | 67421 | 67807 | 387 | 92.05 | 93.28 | 93.28 |
| *rpl22* | 81923 | 82390 | 468 | 62.61 | 85.44 | 87.55 |
| *rpl23* | 84299  149444 | 84580  149725 | 282 | 99.29 | 98.94 | 98.60 |
| *rpl32* | 110870 | 111040 | 171 | 91.81 | 94.56 | 95.65 |
| *rpl33* | 66518 | 66718 | 201 | 89.37 | 93.16 | 92.54 |
| *rpl36* | 78197 | 78310 | 114 | 94.74 | 96.49 | 98.23 |
| *rpoA* | 76592 | 77599 | 1008 | 89.76 | 90.93 | 93.15 |
| *rpoB* | 23473 | 26685 | 3213 | 94.62 | 95.21 | 96.67 |
| *rpoC1* | 20633 | 23467 | 2076 | 94.61 | 94.59 | 96.53 |
| *rpoC2* | 16288 | 20475 | 4188 | 90.41 | 91.66 | 93.99 |
| *rps11* | 77671 | 78087 | 417 | 94.48 | 94.72 | 95.92 |
| *rps12* | 68618  96002  137239 | 68731 96785  138022 | 372 | 82.62 | 98.39 | 98.66 |
| *rps14* | 36184 | 36486 | 303 | 95.71 | 94.72 | 96.70 |
| *rps15* | 120631 | 120903 | 273 | 86.09 | 91.94 | 91.58 |
| *rps16* | 4833 | 5943 | 237 | 92.08 | 94.42 | 97.00 |
| *rps18* | 66884 | 67189 | 306 | 95.75 | 97.71 | 97.39 |
| *rps19* | 82460 | 82738 | 279 | N/A | 91.24 | 93.19 |
| *rps2* | 15369 | 16079 | 711 | 94.09 | 95.50 | 97.33 |
| *rps3* | 81276 | 81938 | 663 | 90.44 | 92.13 | 93.67 |
| *rps4* | 44993 | 45598 | 606 | 93.49 | 96.04 | 95.54 |
| *rps7* | 95481  138076 | 95948  138543 | 468 | 99.36 | 99.15 | 99.36 |
| *rps8* | 78766 | 79170 | 405 | 90.86 | 94.32 | 95.56 |
| *ycf1* | 107179 | 108234 | 5517 | 80.51 | 83.69 | 81.34 |
| *ycf15* | 91850  141926 | 92098  142174 | 249 | 98.39 | 99.33 | 99.20 |
| *ycf2* | 84908  142265 | 91759  149116 | 6852 | 96.44 | 97.01 | 83.71 |
| *ycf3* | 41882 | 43813 | 510 | 95.93 | 95.49 | 97.84 |
| *ycf4* | 59218 | 59772 | 555 | 94.23 | 95.68 | 94.23 |
| *rrn16* | 98687  133847 | 100177  135337 | 1491 | 99.80 | 99.80 | 99.93 |
| *rrn23* | 102585  128629 | 105395  131439 | 2811 | 99.18 | 99.22 | 99.22 |
| *rrn4.5* | 105494  128428 | 105596  128530 | 103 | 100.00 | 100.00 | 100.00 |
| *rrn5* | 105821  128083 | 105941  128203 | 121 | 99.17 | 100.00 | 100.00 |

The percent identity of protein-coding sequences were calculated manually using ClustalW and NCBI BLAST 2 sequences programs.
