# Supplementary material for: The Complete Chloroplast Genome Sequence of the Medicinal Plant Salvia miltiorrhiza
Source: PLoS One. 2013 Feb 27;8(2):e57607. doi: 10.1371/journal.pone.0057607 (PMC3584094; doi:10.1371/journal.pone.0057607)
Supplement: Table S6 — Average pairwise sequence distance of protein-coding genes among the 30 asterid chloroplast genomes. (DOC) [file pone.0057607.s010.doc]

**Table S6.** Average pairwise sequence distance of protein-coding genes among the 30 asterid chloroplast genomes.

| Order | Gene | Region | D | SE | Average length (bp) | Range of length (bp) | Missing taxa | Functional groups |
| --- | --- | --- | --- | --- | --- | --- | --- | --- |
| 1 | *ndhB* | IR | 0.01285 | 0.00040 | 1523 | 1128-1626 |  | NADH dehydrogenase |
| 2 | *rpl2* | IR | 0.02035 | 0.00074 | 825 | 819-831 |  | LSU ribosomal proteins |
| 3 | *psbL* | LSC | 0.02237 | 0.00066 | 117 | 117 |  | Photosystem II |
| 4 | *petG* | LSC | 0.02287 | 0.00087 | 114 | 114 |  | Cytochrom b/f complex |
| 5 | *rps7* | IR | 0.02350 | 0.00143 | 468 | 468 |  | SSU ribosomal proteins |
| 6 | *rpl23* | IR | 0.02432 | 0.00159 | 282 | 279-285 | *Trachelium* (pseudogene) | LSU ribosomal proteins |
| 7 | *psbN* | LSC | 0.02750 | 0.00083 | 132 | 132 |  | Photosystem II |
| 8 | *psbF* | LSC | 0.03044 | 0.00107 | 120 | 120 |  | Photosystem II |
| 9 | *psbZ* | LSC | 0.03209 | 0.00077 | 189 | 189 |  | Photosystem II |
| 10 | *psbA* | LSC | 0.03234 | 0.00064 | 1062 | 1059-1062 |  | Photosystem II |
| 11 | *psbE* | LSC | 0.03279 | 0.00078 | 252 | 252 |  | Photosystem II |
| 12 | *psbD* | LSC | 0.03475 | 0.00063 | 1062 | 1056-1062 |  | Photosystem II |
| 13 | *atpH* | LSC | 0.03637 | 0.00088 | 246 | 246 |  | ATP synthase |
| 14 | *psaA* | LSC | 0.03939 | 0.00074 | 2253 | 2253 |  | Photosystem I |
| 15 | *psbJ* | LSC | 0.04052 | 0.00101 | 123 | 120-123 |  | Photosystem II |
| 16 | *petD* | LSC | 0.04076 | 0.00084 | 485 | 483-525 |  | Cytochrom b/f complex |
| 17 | *ycf3* | LSC | 0.04278 | 0.00095 | 508 | 504-516 |  | ORF of unknown function |
| 18 | *psaB* | LSC | 0.04288 | 0.00082 | 2205 | 2205 |  | Photosystem I |
| 19 | *petN* | LSC | 0.04309 | 0.00157 | 91 | 90-114 |  | Cytochrom b/f complex |
| 20 | *psbC* | LSC | 0.04513 | 0.00083 | 1421 | 1386-1422 |  | Photosystem II |
| 21 | *atpI* | LSC | 0.04707 | 0.00090 | 744 | 735-744 |  | ATP synthase |
| 22 | *petB* | LSC | 0.04936 | 0.00097 | 643 | 489-651 |  | Cytochrom b/f complex |
| 23 | *psbI* | LSC | 0.05151 | 0.00147 | 113 | 111-156 | *Boea* (pseudogene) | Photosystem II |
| 24 | *psbB* | LSC | 0.05154 | 0.00103 | 1527 | 1518-1527 |  | Photosystem II |
| 25 | *ndhJ* | LSC | 0.05258 | 0.00115 | 478 | 477-498 |  | NADH dehydrogenase |
| 26 | *ndhC* | LSC | 0.05329 | 0.00114 | 363 | 363 |  | NADH dehydrogenase |
| 27 | *psaJ* | LSC | 0.05435 | 0.00158 | 133 | 126-135 |  | Photosystem I |
| 28 | *rps14* | LSC | 0.05572 | 0.00132 | 303 | 294-303 |  | SSU ribosomal proteins |
| 29 | *psbT* | LSC | 0.05766 | 0.00143 | 106 | 102-111 |  | Photosystem II |
| 30 | *psbM* | LSC | 0.05901 | 0.00179 | 106 | 105-117 |  | Photosystem II |
| 31 | *atpB* | LSC | 0.05947 | 0.00119 | 1497 | 1497-1500 |  | ATP synthase |
| 32 | *psbK* | LSC | 0.06044 | 0.00140 | 184 | 177-186 |  | Photosystem II |
| 33 | *psbH* | LSC | 0.06105 | 0.00138 | 224 | 222-246 |  | Photosystem II |
| 34 | *rpl14* | LSC | 0.06166 | 0.00148 | 369 | 369-378 |  | LSU ribosomal proteins |
| 35 | *petA* | LSC | 0.06197 | 0.00120 | 963 | 960-963 |  | Cytochrom b/f complex |
| 36 | *atpA* | LSC | 0.06211 | 0.00125 | 1524 | 1503-1527 |  | ATP synthase |
| 37 | *psaC* | SSC | 0.06346 | 0.00142 | 246 | 246 |  | Photosystem I |
| 38 | *rps4* | LSC | 0.06389 | 0.00169 | 607 | 606-612 |  | SSU ribosomal proteins |
| 39 | *rbcL* | LSC | 0.06407 | 0.00121 | 1437 | 1428-1479 |  | Rubisco |
| 40 | *rpl36* | LSC | 0.06414 | 0.00185 | 114 | 114-117 |  | LSU ribosomal proteins |
| 41 | *petL* | LSC | 0.06435 | 0.00171 | 96 | 96 |  | Cytochrom b/f complex |
| 42 | *ycf2* | IR | 0.06506 | 0.00430 | 6652 | 5925-7134 |  | ORF of unknown function |
| 43 | *rps12* | IR/LSC | 0.06538 | 0.00340 | 372 | 357-414 |  | SSU ribosomal proteins |
| 44 | *rps2* | LSC | 0.06546 | 0.00166 | 712 | 711-747 |  | SSU ribosomal proteins |
| 45 | *rpoB* | LSC | 0.06830 | 0.00151 | 3207 | 3183-3216 |  | RNA polymerase |
| 46 | *ndhK* | LSC | 0.06874 | 0.00147 | 721 | 642-855 | *Trachelium* (pseudogene) | NADH dehydrogenase |
| 47 | *ndhH* | SSC | 0.07182 | 0.00137 | 1182 | 1179-1188 |  | NADH dehydrogenase |
| 48 | *rpoC1* | LSC | 0.07289 | 0.00168 | 2068 | 2046-2091 |  | RNA polymerase |
| 49 | *atpF* | LSC | 0.07351 | 0.00168 | 558 | 546-621 |  | ATP synthase |
| 50 | *rps18* | LSC | 0.07383 | 0.00411 | 322 | 306-696 |  | SSU ribosomal proteins |
| 51 | *ndhE* | SSC | 0.07384 | 0.00157 | 306 | 303-306 |  | NADH dehydrogenase |
| 52 | *ndhI* | SSC | 0.07404 | 0.00147 | 505 | 495-513 |  | NADH dehydrogenase |
| 53 | *ycf4* | LSC | 0.07405 | 0.00157 | 555 | 549-555 |  | ORF of unknown function |
| 54 | *ndhG* | SSC | 0.07419 | 0.00140 | 531 | 531 |  | NADH dehydrogenase |
| 55 | *ndhA* | SSC | 0.07612 | 0.00142 | 1091 | 1086-1098 |  | NADH dehydrogenase |
| 56 | *rpl16* | LSC | 0.07683 | 0.00176 | 408 | 360-444 |  | LSU ribosomal proteins |
| 57 | *infA* | LSC | 0.07936 | 0.00433 | 229 | 105-270 | 10 taxa | Translation initiation factors |
| 58 | *rps16* | LSC | 0.08117 | 0.00232 | 259 | 237-294 |  | SSU ribosomal proteins |
| 59 | *atpE* | LSC | 0.08651 | 0.00161 | 403 | 399-423 |  | ATP synthase |
| 60 | *psaI* | LSC | 0.08663 | 0.00195 | 111 | 102-111 |  | Photosystem I |
| 61 | *rps19* | LSC | 0.08837 | 0.00256 | 280 | 279-297 | *Boea* (pseudogene) | SSU ribosomal proteins |
| 62 | *rps8* | LSC | 0.08839 | 0.00179 | 405 | 393-405 |  | SSU ribosomal proteins |
| 63 | *rpl20* | LSC | 0.08900 | 0.00243 | 385 | 351-399 |  | LSU ribosomal proteins |
| 64 | *ndhD* | SSC | 0.09073 | 0.00177 | 1495 | 1152-1542 |  | NADH dehydrogenase |
| 65 | *rps11* | LSC | 0.09121 | 0.00290 | 416 | 402-441 |  | SSU ribosomal proteins |
| 66 | *rpoA* | LSC | 0.09129 | 0.00181 | 1012 | 813-1062 |  | RNA polymerase |
| 67 | *rpl33* | LSC | 0.09457 | 0.00204 | 201 | 195-207 |  | LSU ribosomal proteins |
| 68 | *rpoC2* | LSC | 0.09501 | 0.00195 | 4169 | 4089-4206 |  | RNA polymerase |
| 69 | *rps3* | LSC | 0.10060 | 0.00253 | 660 | 657-699 |  | SSU ribosomal proteins |
| 70 | *cemA* | LSC | 0.10122 | 0.00248 | 690 | 690-693 |  | Miscellaneous proteins |
| 71 | *accD* | LSC | 0.10381 | 0.00230 | 1511 | 1443-1593 | *Jasminum, Trachelium* (pseudogene) | Miscellaneous proteins |
| 72 | *rps15* | SSC | 0.12254 | 0.00246 | 273 | 264-330 |  | SSU ribosomal proteins |
| 73 | *ccsA* | SSC | 0.12321 | 0.00237 | 961 | 939-993 |  | Miscellaneous proteins |
| 74 | *ndhF* | SSC | 0.12624 | 0.00250 | 2225 | 2133-2307 |  | NADH dehydrogenase |
| 75 | *clpP* | LSC | 0.12790 | 0.00693 | 596 | 591-660 | *Trachelium* (pseudogene) | Miscellaneous proteins |
| 76 | *matK* | LSC | 0.14005 | 0.00280 | 1527 | 1503-1578 |  | Maturase |
| 77 | *rpl32* | SSC | 0.14362 | 0.00451 | 170 | 162-222 |  | LSU ribosomal proteins |
| 78 | *rpl22* | LSC | 0.15040 | 0.00312 | 471 | 360-540 |  | LSU ribosomal proteins |
| 79 | *ycf1* | IR-SSC | 0.27611 | 0.00996 | 5595 | 5040-6411 | *Ageratina* (pseudogene) | ORF of unknown function |
| 80 | *ycf15* | IR | 0.40601 | 0.06945 | 204 | 135-303 | 17 taxa | ORF of unknown function |

D and SE indicate average sequence distances and standard errors, respectively.
